# The Role of Shear Stress in Arteriovenous Fistula Maturation and Failure: A Systematic Review

**DOI:** 10.1371/journal.pone.0145795

**Published:** 2015-12-30

**Authors:** Leonard D. Browne, Khalid Bashar, Philip Griffin, Eamon G. Kavanagh, Stewart R. Walsh, Michael T. Walsh

**Affiliations:** 1 Centre for Applied Biomedical Engineering Research (CABER), Department of Mechanical, Aeronautical and Biomedical Engineering, Materials and Surface Science Institute, The Health Research Institute, University of Limerick, Limerick, Ireland; 2 Department of Vascular Surgery, Limerick University Hospital, Dooradoyle, Limerick, Ireland; 3 Department of Surgery, National University of Ireland, Galway, Ireland; University of Washington, UNITED STATES

## Abstract

**Introduction:**

Non-maturation and post-maturation venous stenosis are the primary causes of failure within arteriovenous fistulae (AVFs). Although the exact mechanisms triggering failure remain unclear, abnormal hemodynamic profiles are thought to mediate vascular remodelling and can adversely impact on fistula patency.

**Aim:**

The review aims to clarify the role of shear stress on outward remodelling during maturation and evaluate the evidence supporting theories related to the localisation and development of intimal hyperplasia within AVFs.

**Methods:**

A systematic review of studies comparing remodelling data with hemodynamic data obtained from computational fluid dynamics of AVFs during and after maturation was conducted.

**Results:**

Outward remodelling occurred to reduce or normalise the level of shear stress over time in fistulae with a large radius of curvature (curved) whereas shear stress was found to augment over time in fistulae with a small radius of curvature (straight) coinciding with minimal to no increases in lumen area. Although this review highlighted that there is a growing body of evidence suggesting low and oscillating shear stress may stimulate the initiation and development of intimal medial thickening within AVFs. Further lines of evidence are needed to support the disturbed flow theory and outward remodelling findings before surgical configurations and treatment strategies are optimised to conform to them. This review highlighted that variation between the time of analysis, classification of IH, resolution of simulations, data processing techniques and omission of various shear stress metrics prevented forming pooling of data amongst studies.

**Conclusion:**

Standardised measurements and data processing techniques are needed to comprehensively evaluate the relationship between shear stress and intimal medial thickening. Advances in image acquisition and flow quantifications coupled with the increasing prevalence of longitudinal studies commencing from fistula creation offer viable techniques and strategies to robustly evaluate the relationship between shear stress and remodelling during maturation and thereafter.

## Introduction

Hemodialysis is the treatment modality of choice for patients with end stage renal disease (ESRD). Adequate and efficient hemodialysis requires a reliable vascular access which is easily accessible and provides consistently high flow rates greater than 600 ml/min [1,2]. Arteriovenous fistulae (AVFs) are the preferred access choice due to lower infection and stenosis rates. However, they are prone to complications during remodelling and have a high incidence of primary failure during this process [2]. Thrombotic occlusion arising from aggressive intimal hyperplasia (IH) and impaired remodelling leading to reduced flow rates at the access site, are the two major causes of patency loss [2–5]. Complex AVF hemodynamics are believed to provide a stimulus for remodelling and IH related failure [6,7]. Computational fluid dynamics (CFD) allows for the study of hemodynamics within multiple vasculatures as it can approximate analytically complex flow fields. CFD can also calculate hemodynamic parameters derived from the flow field such as shear stress. Owing to the improving resolution of medical imaging and the ability to decompose these images into CFD models, modelling hemodynamics of realistic patient geometries is possible [8].

The review aims to clarify the role of shear stress on outward remodelling during maturation and evaluate the evidence supporting theories related to the localisation and development of intimal hyperplasia within AVFs.

## Methods

### 2.1 Search strategy

A systematic search was conducted on the PubMed and Google scholar database for articles published online before 1/1/2015 whose title/abstract contained the following sequence of keywords as outlined in Table 1.

**Table 1:** Search string for PubMed which produces 131 results with a filter for publication date to 2015/01/01.

Strategy	#	Search Terms
Population	#1	(Vascular access[Title/Abstract] OR Arteriovenous fistul*[Title/Abstract] OR Arteriovenous Shunt[Title/Abstract] OR AVF[Title/Abstract] OR arteriovenous graft[Title/Abstract] OR anastomos*[Title/Abstract])
Intervention	#2	(CFD[Title/Abstract] OR computational fluid dynamics[Title/Abstract] OR numerical*[Title/Abstract] OR simulat*[Title/Abstract] OR in-situ[Title/Abstract] OR comput*[Title/Abstract] OR calcul*[Title/Abstract])
Comparison	#3	(hemodynamic*[Title/Abstract] OR shear stress[Title/Abstract] OR WSS[Title/Abstract] OR biomechanical forces[Title/Abstract] OR stress[Title/Abstract] OR mechanical[Title/Abstract])
Outcomes	#4	(intima media thickness[Title/Abstract] OR stenos*[Title/Abstract] OR intimal hyperplasia[Title/Abstract] OR smooth muscle cell migration[Title/Abstract] OR SMC remodelling[Title/Abstract] OR vascular remodelling[Title/Abstract] OR endothelial cell[Title/Abstract] OR vascular access dysfunction[Title/Abstract] OR MMP[Title/Abstract] OR maturation[Title/Abstract] OR patholog*[Title/Abstract] OR mature[Title/Abstract] OR IMT[Title/Abstract] OR IH[Title/Abstract] OR intima-media[Title/Abstract])
	#5	NOT (coronary OR pulmonary OR catheter)
	#6	#1 AND #2 AND #3 AND #4 AND #5

Acronyms: WSS = Wall Shear Stress, MMP = Matrix Metalloprotease, IH = intimal hyperplasia, IMT = Intima Media Thickening

### 2.2 Eligibility criteria

**Fig 1:**
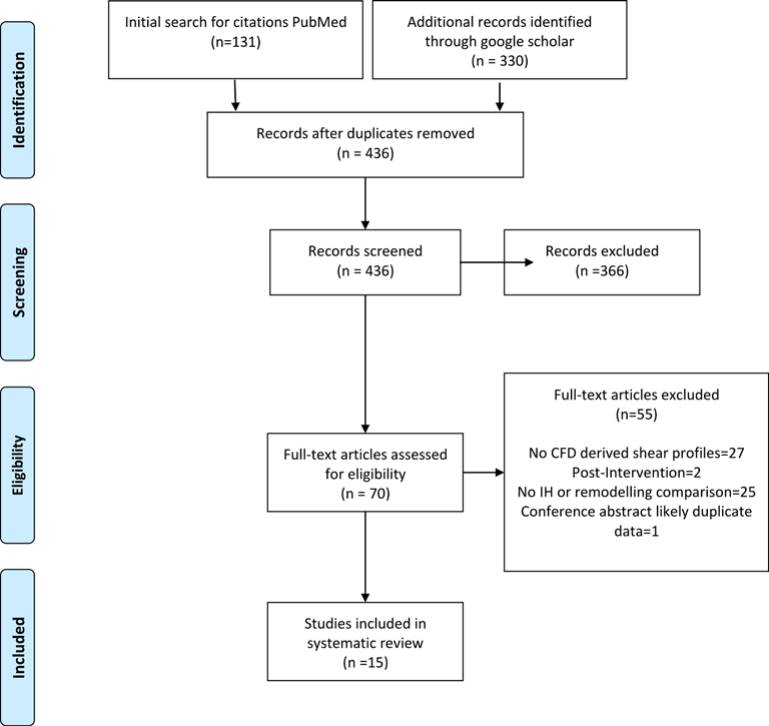
PRISMA diagram: Flow chart of the strategy used to select articles for review.

The titles and abstracts of all potentially suitable studies were inspected, articles meeting the inclusion criteria were retrieved and reviewed by review authors (LB, MW).The results of the process are illustrated in [Fig 1]. Included in the review were:

- Studies which analysed blood flow in one or more vascular access geometries which are anatomically realistic and were acquired by a relevant imaging modality
- Studies in which the localisation of stenotic lesions or adaptive responses were based on the presence of an established marker of disease or remodelling
- Studies in which results were compared against lesion data from a companion paper from the same group, or from a cited article from a different research group for the same species
- Studies which discussed the relationship between blood flow or shear stress on vascular access remodelling or the formation of intimal hyperplasia

Excluded from the review were:

- Studies which did not attempt to compare hemodynamic results in detail with the localisation of lesions or adaptive responses in the relevant species
- Studies in which geometries were acquired after interventional revisions
- Studies which superimposed stenosis by ligation or other means to alter flow as these studies may not replicate the normal initiation of the disease
- Studies which utilised idealised geometries

### 2.3 Data extraction

Data from each article was extracted by two review authors (LB, MW) and were compared for consistency of data extraction; any disagreement was discussed with an additional author. The following information regarding AVF characteristics were recorded for maturation studies: Time post creation, image modality, radius of curvature, species, site location, variation of shear stress, flow rate, cross sectional area (CSA) and intima-media thickness (IMT). For IH studies the following were extracted: Time post creation, patterns of shear stress related parameters, biological markers, image modality, experimental modality and IMT.

### 2.4 Selected literature

Fifteen articles were selected from an original set of 436. Removing duplicates, reviews and articles that did not relate to hemodynamics within AVFs left 70 articles. A further 25 were discarded because they did not compare their hemodynamic results with the localisation of lesions or adaptive responses in relevant species. 27 articles were removed as they did not provide shear stress computed data. An additional 2 articles were removed as they were conducted on geometries pre and post interventional treatment. Finally, 1 article was removed as it was likely a duplicate conference preceding. After screening, 15 articles which compared remodelling or IH data with shear stress metrics remained as outlined in Table 2.

**Table 2:** Reviewed articles in chronological order, with the affiliation of the corresponding author, species and AVF configuration.

REF	Title	Year	Affiliation of Corresponding Author	Species	Fistula
8	Measurement of hemodynamic and anatomic parameters in a swine arteriovenous fistula model	2008	University of Cincinnati	Porcine	Femoral artery and femoral vein AVF
15	Longitudinal assessment of hemodynamic endpoints in predicting arteriovenous fistula maturation	2013	University of Cincinnati	Porcine	Femoral artery and femoral vein AVF
16	Influence of temporal variation in wall shear stress on intima-media thickening in arteriovenous fistulae	2012	University of Cincinnati	Porcine	Femoral artery and femoral vein AVF
17	Vascular remodeling in autogenous arterio-venous fistulas by MRI and CFD	2013	University of California	Human	Brachiocephalic (n = 2) Brachiobasillic (n = 1)
18	Serial analysis of lumen geometry and hemodynamics in human arteriovenous fistula for hemodialysis using magnetic resonance imaging and computational fluid dynamics	2012	University of Utah	Human	Brachiocephalic
19	Hemodynamic wall shear stress profiles influence the magnitude and pattern of stenosis in a pig AV fistula	2008	University of Cincinnati	Porcine	Femoral artery and femoral vein AVF
20	Numerical and experimental study of blood flow through a patient-specific arteriovenous fistula used for hemodialysis	2010	Universite de Technologie de Compiegne	Human	Brachiocephalic
21	Investigations into the relationship between hemodynamics and vascular alterations in an established arteriovenous fistula	2007	Universite de Technologie de Compiegne	Human	Brachiocephalic
22	Incomplete restoration of homeostatic shear stress within arteriovenous fistulae	2013	University of Washington	Human	Radiocephalic (n = 2) Brachiocephalic(n = 2)
23	Wall shear stresses remain elevated in mature arteriovenous fistulas: a case study	2011	University of Limerick	Human	Radiocephalic AVF
31	Realistic temporal variations of shear stress modulate MMP-2 and MCP-1 expression in arteriovenous vascular access	2009	University of Limerick	Human	Radiocephalic
32	New Techniques for Determining the Longitudinal Effects of Local Hemodynamics on the Intima‐Media Thickness in Arteriovenous Fistulae in an Animal Model	2013	University of Cincinnati	Porcine	Femoral artery and femoral vein AVF
41	Transitional flow at the venous anastomosis of an arteriovenous graft: potential activation of the ERK1/2 mechanotransduction pathway	2003	The University of Illinois at Chicago	Canine	Femoral artery to Femoral vein graft
52	Numerical simulation of the fluid structure interactions in a compliant patient‐specific arteriovenous fistula	2014	Universite de Technologie de Compiegne	Human	Radiocephalic
53	Effects of wall distensibility in hemodynamic simulations of an arteriovenous fistula	2013	University of Washington	Human	Radiocephalic

Eight articles focused on the role or distribution of shear stress within maturing or mature AVFs. Five articles focused on the roles of shear stress metrics or hemodynamic metrics in relation to IH formation. Two articles focused on the influence of distensibility on the shear stress distribution and its influence on IH. The selected articles originated from 7 institutions and studied human & porcine models to assess maturation and intimal hyperplasia formation with the addition of a canine model for the later. The brachiocephalic, radiocephalic and brachiobasillic fistula were constructed in humans. Anastomoses between the femoral artery and vein and the aorta and iliac vein were the configurations created in porcine models.

## Maturation

Maturation is the remodelling process whereby a fistula becomes suitable for cannulation. AVFs are assessed for non-maturation after 4–6 weeks; mature AVFs should have a diameter of 6mm, be less than 6mm below the skin surface and have a flow rate greater than 600 ml/min [9].

**Fig 2:**
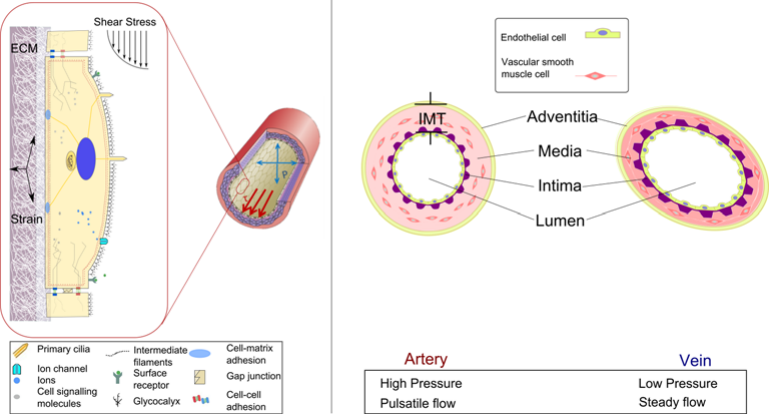
A section of an artery wall shows the endothelial cells that form the inner lining and align longitudinally in the direction of the flow. Pressure (P) acts normal to the vessel wall, which results in circumferential stretching of the vessel wall. Shear stress (τ) is parallel to the vessel wall and is exerted longitudinally in the direction of blood flow. The intima, media and adventitia layers of an artery and vein are shown. Vascular smooth muscle cells form the outer layers and align circumferentially. IMT refers to intima media thickness.

Endothelial cells (ECs) which line the internal surface of a blood vessel are important mediators of maturation. ECs are continually exposed to shear stress, compression via blood pressure and to tension from strain in the extracellular matrix (ECM) as shown in [Fig 2]. Each of these factors is known to modulate intracellular signalling pathways and gene expression. Variations of these mechanical factors from the normal level can alter EC function and stimulate remodelling [10–12]. Exposure of a vein to the arterial environment during AVF creation is a paradigm of such.

**Fig 3:**
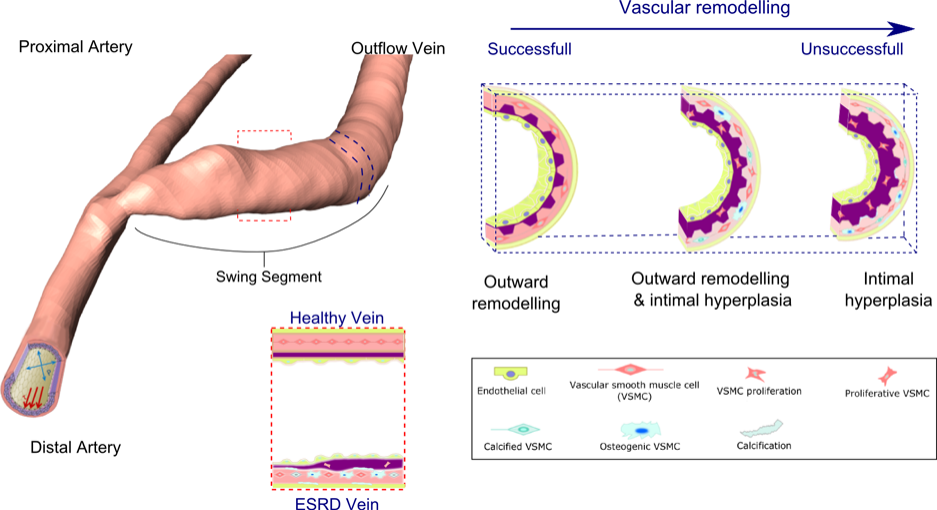
Typical geometry of an arteriovenous fistula is shown with the swing segment highlighted; the dashed blue line highlights a cross section of the vein for which various vascular remodelling responses within the venous segment of an AVF are shown for a healthy vein and an ESRD vein.

AVF formation involves directly connecting a high pressure pulsatile flow conduit (artery) to a low pressure steady flow conduit (vein). The resulting pressure gradient results in an immediate increase in flow in both the artery and vein and the resulting hemodynamics initiates a vascular remodelling response within both vessels. Pressure in the venous segment rises upon AVF creation and remains relatively constant during the time course of remodelling and thereafter. The increase in pressure is known to stimulate vascular smooth muscle cell (VSMC) proliferation and induce moderate medial thickening over time [13]. Remodelling in maturing AVFs is primarily characterised by eccentric medial hypertrophy resulting from increased circumferential tension due to flow mediated dilation rather than the elevated pressure alone [14]. Dilation is induced by high levels of shear stress, resulting from elevated flow. The level of shear stress is also known to regulate the intimal layer with a well-established inverse correlation between intimal thickness and shear stress observed during venous and arterial adaption [13]. One can conjecture that the balance between dilation and intimal medial thickening will determine whether a favourable outward hypertrophic remodelling or unfavourable inward hypertrophic remodelling response occurs [6], these remodelling responses are illustrated in [Fig 3].

Endothelial cells are finely primed to sense variations in shear stress and respond accordingly. Therefore, knowledge of shear stress patterns within AVFs as they progress to maturity could help predict the molecular and structural responses that occur during remodelling, thereby identifying critical linkages and targets for therapeutic strategies and intervention.

### 3.1 Longitudinal studies

A longitudinal study is a correlational research study that involves repeated observations of the same variables over long periods of time. A number of studies adopted this approach to monitor shear stress and structural changes within an AVF during its maturation period. For this review AVFs were categorised as either straight or curved based on a qualitative assessment of the radius of curvature of the swing segment to assess the variation of flow rate and shear stress on outward remodelling. Curved AVFs were categorised by a large radius of curvature, identified by the gradual bend of the fistulas swing segment. Whereas, straight AVFs were categorised by a small radius of curvature, identified by an abrupt bend of the swing segment.

For curved AVFs the flow rate within the venous segment increases during the first 6 weeks [Table 3]. Cross sectional area increases to reduce the level of shear stress to its physiological range. Intimal medial thickening increases progressively during this period. The increase in area varies along the length of the venous segment due to the non-uniform distribution of shear stress in the segment. After 6 weeks flow rate decreases and cross sectional area continues to increase to reduce the level of shear stress. This trend may fluctuate about this time point in a larger cohort due to higher levels of heterogeneity. Nonetheless, high shear stresses are found to persist near the anastomosis. Further away from the anastomosis shear stress is found to decrease towards the physiological range.

**Table 3:** Variation of experimental conditions and outcomes for AVF with curved configurations i.e. time of analysis, variation of flow rate, shear stress, lumen cross sectional area (CSA) and intima media thickness (IMT).

Stage	Author	Methodology	Species & (location)	Time post formation	Flow rate	Shear stress level	CSA	IMT
<6 wks.	Rajabi-Jagahrgh et al [15,16].	CT angiography Ultrasound CFD Histological analysis	Porcine (Femoral artery and vein) n = 3	2 days	-	-	-	-
				7 days	↑	↓	↑	↑
				28 days	↑	↓	↑	↑
	Sigovan et al [17].	Magnetic resonance angiogram CFD	Human (Brachiocephalic) n = 2	5 days	-	-	-	-
				1 mo.	↑	↓	↑	X
>6 wks.				3 mo.	↓	↓	↑	X
	He et al [18].	Magnetic resonance angiogram CFD	Human (Brachiocephalic) n = 1	4 mo.	-	-	-	-
				5 mo.	↓	↓	↑	X
				7 mo.	↓	↓	↑	X

X no data available; ↑ Increase; ↓ Decrease;—Initial time point of analysis

For straight AVFs the flow rate was found to increase during the first 6 weeks, cross sectional area did not significantly change during this period and the level of shear stress was found to increase [Table 4]. Intimal medial thickness was also found to progressively increase during this period. High shear stresses were found to extend into and occupy a larger proportion of the venous segment compared to curved configurations.

**Table 4:** Variation of experimental outcomes for AVF with straight configurations.i.e. time of analysis, variation of flow rate, shear stress, lumen cross sectional area (CSA) and intima media thickness (IMT).

Stage	Author	Methodology	Species & (location)	Time post formation	Flow rate	Shear stress level	CSA	IMT
<6 wks.	Rajabi-Jagahrgh et al [15,16].	CT angiography Ultrasound CFD Histological analysis	Porcine (Femoral artery and vein) n = 3	2 days	-	-	-	-
				7 days	↑	↑	↔	↑
				28 days	↑	↑	↔	↑
	Sigovan et al [17].	Magnetic resonance angiogram CFD	Human (Brachiobasillic) n = 1	5 days	-	-	-	-
				1 mo.	↑	↑	↔	X
>6 wks.				3 mo.	↑	↑	↗	X

X no data available; ↑ Increase; ↓ Decrease;—Initial time point of analysis; ↔ no significant change; ↗ minor increase)

Rajabi-Jagahrgh et al [15] found that intima media thickness increases progressively for both configurations with a significant difference between the two configurations at 28 days with greater thickening within the curved configuration. An earlier study by Krishnamoorthy et al [19] found an inverse correlation between shear stress and IMT. Hence, as lower levels of shear stress occupy the curved configuration greater thickening may be induced.

### 3.2 Single time point studies

A number of computational studies were conducted at single time points in mature patent fistulae in an attempt to characterize the hemodynamics of these patent accesses. All of which found that shear stress was not homogeneously distributed over the vessel with high non homeostatic shear stresses persisting at the arterial curvatures feeding the anastomosis, within the anastomosis junction and within the swing segment of the vein just after the anastomosis due to the jet velocity striking the wall. Further away from the anastomosis shear stresses were within the physiological range [Table 5].

**Table 5:** Species and location and shear stress distribution of mature AVFs assessed at single time points.

Author	Methodology	Species & (location)	Time post formation	Shear stress level		
				AJ	SS	V
Kharboutly et al [20,21].	CT angiography CFD	Human (Brachiocephalic) n = 1	20 yrs.	High	High	Normal
McGah et al [22].	Ultrasound CFD	Human (Radiocephalic) n = 2	7.6 yrs.	High	High	Normal
			2.0 yrs.	High	High	Normal
		(Brachiocephalic)n = 2	3.3 yrs.	High	High	Normal
			2.2 yrs.	High	High	Normal
Carrol et al [23].	MRI Ultrasound CFD	Human (Radiocephalic) n = 1	>1yr.	High	High	Normal

AJ Anastomotic Junction; SS Swing Segment; V Vein

## Intimal Hyperplasia

Intimal hyperplasia (IH) has been cited as the underlying cause of stenotic lesion formation within AVFs. Unlike atherosclerosis which is a chronic, inflammatory, fibroproliferative disease of the vascular wall that gradually occurs in time [24], IH is a rapid adaptive response to injury of the endothelium by surgical, hemodynamic, immune or metabolic stresses. The events leading to IH can be divided into an (i) inflammatory (ii) proliferation and (iii) remodelling phase as outlined in Table 6. IH is characterised by VSMC proliferation, followed by VSMC and intimal growth which can diminish the vascular lumen [4,25]. IH primarily comprises of α-smooth muscle actin (α-SMA) positive cells, extracellular matrix proteins and cytokines such as platelet-derived growth factor, transforming growth factor-β and endothelin within the intima and media of the vein [26,27]. The majority of the α-SMA positive cells in the intimal lesions exhibit a myofibroblast or synthetic VSMC phenotype [28–30]. The source and action of the main cytokines, proteinases and growth factors involved in IH formation are summarised in Table 7.

**Table 6:** Events leading to intimal hyperplasia.

(i)	Endothelium injury
	↓
	Platelet adhesion
	↓
	Aggregation and activation of platelets and inflammatory cells at the site of endothelial injury
	↓
(ii)	VSMC proliferation and migration to intima
	↓
	Re-endothelialisation of the injured site
	↓
(iii)	Intimal thickening via secretion of ECM composed of elastin, collagens, glycoproteins and proteoglycans
	↓
	Adventitial fibroblasts migrate into intima and differentiate into myofibroblasts

**Table 7:** Main proteinases, cytokines and growth factors involved in IH formation.

		Source	Action
**Growth Factor**	PDGF	Platelets, ECs, VSMCs	VSMC proliferation and migration
	TGF-B	ECs, VSMCs	VSMC proliferation
	IGF-1	VSMCs	
	bFGF	VSMCs	
	VEGF	ECs	Endothelisation
**Cytokines**	MCP-1	Macrophages, VSMCs, ECs, Fibroblasts	Monocyte recruitment
	IL-1, IL-6	Leucocytes, macrophages, VSMCs, ECs, Fibroblasts	Neutrophil and monocyte recruitment
**Proteinases**	MMP-2 MMP-9	ECs, VSMCs, Macrophages	ECM degradation and reorganisation VSMC proliferation and migration Fibroblast migration
	TIMPs	ECs, VSMCs, Macrophages	Reduced proliferation and migration

PDGF, platelet-derived growth factor; bFGF, basic fibroblast growth factor; IGF, insulin-like growth factor; TGF, transforming growth factor; VEGF, vascular endothelial growth factor; IL, interleukin; MCP-1 monocyte chemoattractant protein 1 MMPs; Matrix metalloproteinases, TIMPs; tissue inhibitors of MMPs

### 4.1 Theories

There are a number of theories related to the influence of shear stress parameters on the development of IH within AVFs. Table 8 outlines the findings from the selected articles supporting magnitude and gradient based shear stress parameters. Definitions for these parameters are provided within S1 Table. All configurations were included to establish the role of various shear stress metrics on inward remodelling.

**Table 8:** Overview of the different theories reviewed on IH within AVFs and their experimental conditions, i.e. time of analysis, shear parameter measured and effect on biological markers and intima media thickness.

Theory	Author	Methodology	Time post formation	Shear Parameter	Biological markers	IMT
High Shear	Carroll et al [31].	CFD Cone & plate	1.5 hr.	High WSS	↑MMP-2 ↑MCP-1	X
			12 hr.			
	Carroll et al [23].	CFD	X	↑WSS ↑WSSG	X	X
Low shear	Krishnamoorthy et al [19].	CFD Histological analysis CT angiography Micro MRI	42 days	Low WSS	X	Max IMT
			42 days	High WSS	X	Min IMT
Low shear & OSI	Rajabi-Jagahrgh et al [32].	CFD * Histological analysis	2 days	Low WSS & high OSI		
			28 days		X	Max IMT
	Kharboutly et al [21].	CFD CT angiography	20 yrs.	OSI	No association to calcified plaque	X
Temporal WSS gradient	Kharboutly et al [21].	CFD CT angiography	20 yrs.	High TWSSG	Strongest association to calcified plaque	X

X no data available; ↑ Increase; ↓ Decrease.

* CFD results at 2 days compared against Histological analysis at 28 days.

#### 4.1.1. High shear stress

Carroll et al [23,31] suggested that high shear stress could induce the upregulation of MCP-1 and MMP-2 and initiate IH in regions of high shear which could denude ECs and expose a thrombogenic surface. However, high shear stresses have been found to persist in mature patent fistulae’s ranging from 2 to 20 yrs. post creation. [20–22]. Whether an unfavourable threshold of high shear stress exists has yet to be quantified.

#### 4.1.2. Low and Oscillatory shear stress

Disturbed flow accounts for a pattern of flow that is non-uniform and irregular; including recirculation eddies and changes in direction with time and space [33]. The combination of low and oscillatory shear stress has been utilised as an indicator of disturbed flow within AVFs. The low shear stress results were paired against intimal medial thickening. Krishnamoorthy et al [19] compared circumferential averages of IMT at selective locations against averaged shear stress and reported an inverse correlation between shear stress and IMT. Rajabi-Jagahrgh et al [32] compared the circumferential distribution of shear stress and IMT at selective locations. Sites of low shear and high oscillating shear index at early time points correlated to maximum IMT at later time points. However, IMT was also observed in a location downstream in a region of higher shear and low OSI. This suggests that multiple aspects of shear stress may stimulate intima-media thickening or that multidirectional aspects of disturbed flow might be described more accurately with metrics such as transverse wall shear stress (transWSS) rather than OSI alone. Nonetheless, the low and oscillating shear stress theory associated with IMT may not be as a robust as previously taught.

#### 4.1.3. Temporal gradient of shear stress

The study by Kharboutly et al [20,21] reported a strong association with high temporal wall shear stress gradient (TWSSG) and locations of calcified plaque compared to low WSS and OSI and concluded that this parameter may be an important determinant of endothelial cell function and plaque formation.

#### 4.1.4. Turbulence

Transitional to turbulent flow has been found to cause EC elongation similar to laminar flow but a loss of EC alignment due to the fluctuating shear stress component [34,35]. A significant reduction in nitric oxide (NO) production, an important inhibitor of inflammation and VSMC proliferation, has also been reported *in-vitro* on EC cultured compliant tubes [36]. Therefore, the presence of transitional flow can disrupt normal function of ECs within AVFs.

Early studies by Fillinger et al [37,38] reported a direct correlation with Reynolds number and IH and hypothesised that turbulence leads to IH via the transfer of kinetic energy to the vessel wall in the form of vein wall vibration within AVFs. However, subsequent studies were unable to validate the theory. This was accredited to computational assumptions such as steady flow, rigid walls and flow split which could bias results. The latter was found to contribute significantly to promoting a transitional regime [39,40].

Loth et al [41] reported that fluctuating shear stress overlapped with areas of vein wall vibration coinciding with regions of elevated extracellular regulatory kinases (ERK1/2) which are linked to the upregulation of PDGF and TGF following vessel injury. None of the previous studies which quantitatively compared shear stress parameters and IH noted a transitional regime. Most determined that peak Reynolds numbers did not reach the critical value for turbulence in a straight pipe (Re>2000) and therefore the laminar flow model was likely to be adequate. However, unstable physiological flow has been simulated by a number of studies attempting to resolve characteristics of the flow field within an AVF [22,42,43]. Ene-Iordache et al [43] have highlighted that transitional to turbulent flow can lead to fluctuations and multidirectional disturbed flow; aspects which have been obscured by under-resolved simulations. The presence of these instabilities could account for the inconsistency between patterns of IMT and hemodynamic metrics observed by Rajabi-Jagahrgh et al [32].

## Hemodynamics of AVFs Were Assessed at Different Sites and Stages of Maturation

### 5.1 Maturation studies

**Fig 4:**
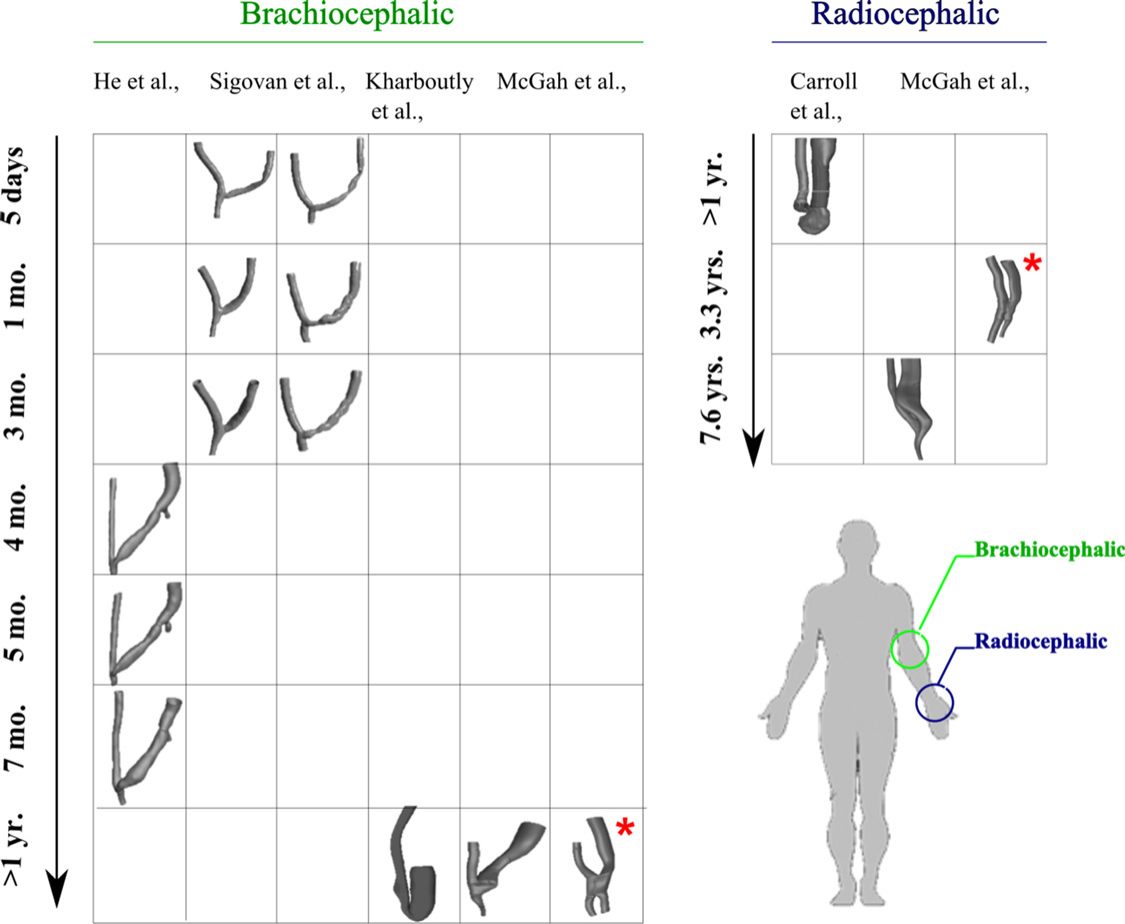
Reconstructions of curved brachiocephalic & radiocephalic fistulae at different longitudinal time points. * indicates a side to side configuration all other AVFs were configured in an end to side manner.

Remodelling alters lumen geometry and significantly alters the distribution and level of shear stress. Hence, the time points at which studies were conducted will impact the geometry and pattern of shear stress. Therefore, the validity of inter-study means for area, flow, Reynolds number and shear stress depends on the time of analysis, location of access site, surgical configuration of the AVF and the accuracy of its reconstruction. [Fig 4] outlines the various reconstructions of patient specific brachiocephalic fistulae and radiocephalic fistulae against the times at which resulting hemodynamics were assessed. The majority of AVFs had an end-side configuration with two AVFs configured in a side to side manner. From visual comparisons it is evident that adequate dilation is a precursor of a mature fistula.

### 5.2 IH studies

During the initial six week period following AVF formation it is expected that high shear stress will promote dilation and subsequent remodelling in the form of expansion and medial hypertrophy. Elementary factors such as MMPs involved in outward remodelling are also involved in IH formation [44–47]. The early expression of MMPs may be mediated by nitric oxide production in response to increased flow and shear stress, the subsequent vasodilation response increases circumferential stretch and thickens the medial layer [11,48]. Therefore, increases in IMT will be attributed in part to medial hypertrophy and will account for the expression of certain genes and proteases which are involved in both hypertrophic remodelling and intimal hyperplasia.

## Species Varied Amongst Studies

Porcine and human models were included in the review as it is known that these models have similar vascular responses to injury with a direct correlation between the magnitude of vascular injury and the amount of intimal hyperplasia. Porcine models can adequately develop intimal hyperplasia over a short period, in contrast to canine or murine models [28]. Intimal thickening within porcine models conformed to the low and oscillatory shear stress theory. Whether this theory is transferrable to ESRD patients remains to be determined as lesion patterns are known to vary amongst species and at different age grades.

## Intimal Hyperplasia Was Classified by Various Modalities and Characterised at Different Pathological Stages

The progression of IH was determined by the identification of one or more proteases, genes or growth factors that lead to its development. Measurements of geometrical characteristics of the vessel wall were also undertaken and IH was also identified by contrast thresholds.

### 7.1 Histological analysis

Carroll et al [31] utilised a cone and plate bioreactor and real time reverse-transcriptase PCR to assess the expression of proteases and cytokines following the onset of flow similar to an AVF environment. The overlap of certain genes and growth factors involved in IH and outward remodelling make it difficult to determine which pathological response will be initiated when these factors are expressed following the onset of flow.

A number of studies conducted histological analysis on porcine models over a longitudinal period. This allowed IMT to be assessed through the use of Hematoxylin and eosin (H&E). This staining makes it difficult to identify the interface between the intimal and medial layers. The subsequent use of Verhoeff’s van Gieson (VVG), PCNA and α-SMA stain have been shown to define these layers [49,50].

### 7.2 Geometrical characteristics

Three studies [15,16,32] utilised IMT to assess pathological change in response to hemodynamic conditions. The technique involves measuring the length or thickness between the outer boundary of the vessel and the intimal layer. This technique makes it difficult to delineate between the intimal and medial layers and subsequent amount of thickening. The time point of analysis will be important as studies conducted within six weeks of formation which assed IH by such means will account for progressive changes that are in part due to outward remodelling.

Therefore, the data processing technique chosen must be quantitatively robust to distinguish layers and capture the initiation or development of IH. The method adapted by Rajabi-Jagahrgh et al [32] in which the circumferential distribution of shear stress and IMT were compared is a viable option. A radiopaque marker was sutured along the fistula providing a fixed local reference point to orient cross-sectional slices. This marker is defined as zero degrees from the luminal centre. Shear stress metrics and IMT are calculated and compared from 0–360 degrees along the circumference of the slice to provide a point by point comparison. As the suture is a fixed local reference point, results between different time points can also be compared. High resolution ultrasound and Micro MRI may offer valid non-invasive imaging approaches to distinguish between the layers but are currently unable to identify its composition [32,51].

### 7.3 Contrast thresholds

Kharboutly et al [20,21] utilised CT angiography to visualise and reconstruct the lumen and calcified plaques within a patent 20 yr. old mature AVF. Calcification zones were identified by their high contrast value within the vascular lumen. Virtual removal of the plaques was carried out and the localisation of calcified plaque against shear stress derived parameters **w**as analysed. The authors acknowledged that the imaging technique made it difficult to differentiate between IH regions and atherosclerotic plaque regions.

## Data Reduction

**Fig 5:**
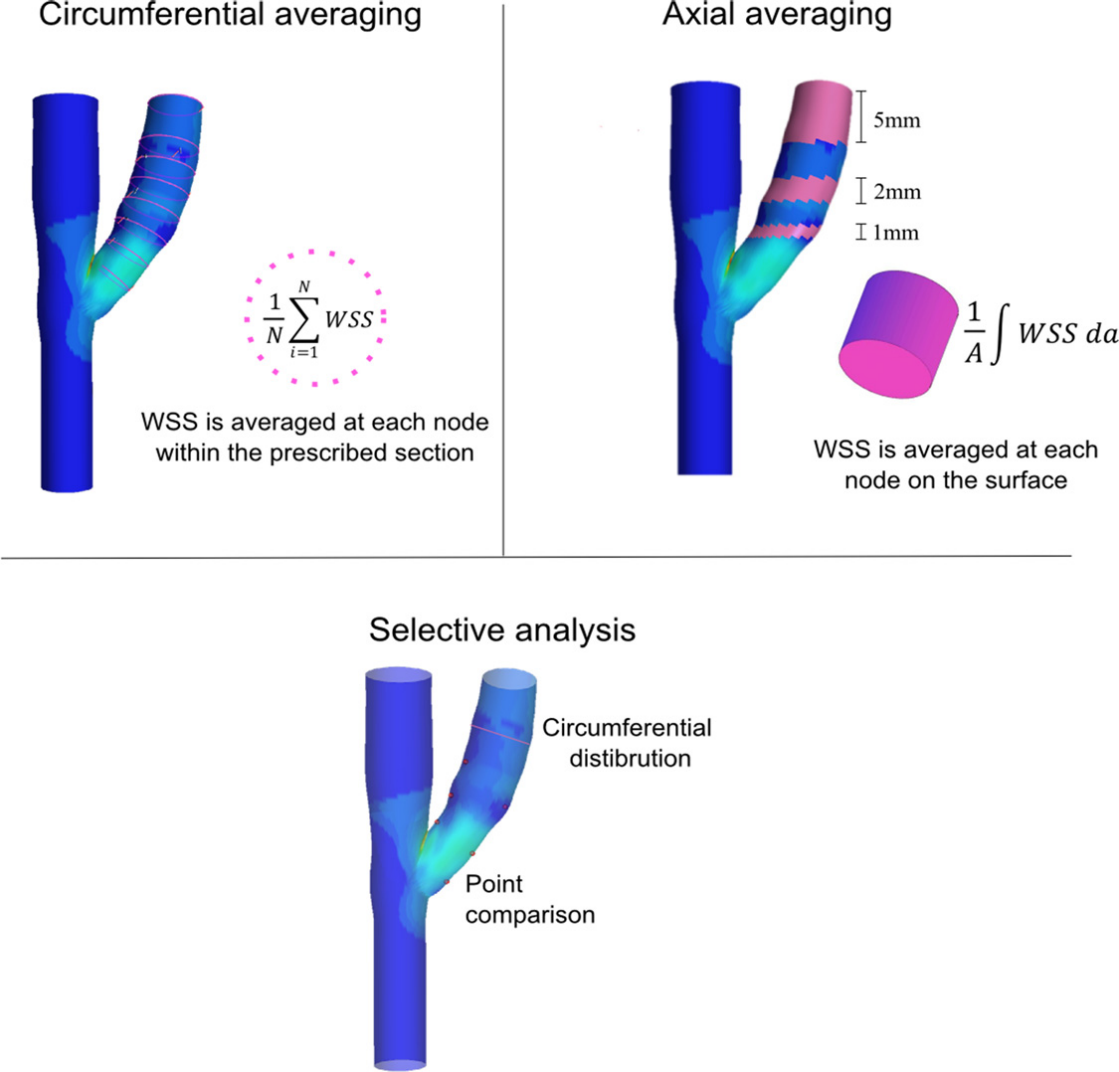
Schematic overview of the various data-processing methods employed in the reviewed articles.

A number of data processing methods were employed to assess the influence of shear stress related parameters on maturation or IH lesion formation [Fig 5]. The choice of technique utilised to compare hemodynamic maps with pathological remodelling can potentially limit the fidelity of conclusions drawn.

### 8.1 Circumferential averaging

- Rajabi-Jagahrgh et al [15] circumferentially averaged TAWSS, IMT and lumen area at 20 different cross sections along the venous segment and attained an overall average of each. They reported that the reduction in shear stress over time was found to have a statistically significant effect on the increase of lumen area over time (r = -0.77, p<0.05).
- He et al [18] circumferentially averaged TAWSS, WSSG and OSI and lumen area at 1 mm intervals; a significant increase in lumen area between each time point was recorded, no other statistical relationships were presented.
- Krishnamoorthy et al [19] circumferentially averaged shear stress at 4 different locations and reported an inverse correlation between WSS and luminal stenosis.

The lumen does not encounter uniform levels of shear stress especially in areas of curvature such as the swing segment of an AVF. Therefore, circumferentially averaging obscures the influence of vessel curvature on IMT and shear stress

### 8.2 Axial Averaging

Axial averages of TAWSS were acquired at 5 mm intervals by McGah et al [22] to compare the results with the value of shear stress determined by Pouseuille’s flow equation which was found to underestimate shear stress. Sigovan et al [17] also utilised a similar technique by averaging the TAWSS on the surface of the venous outflow between two planes from the suture line to the venous outlet. The distribution of shear stresses was found to decrease over time for both brachiocephalic patients. No correlation or significance probability was reported for the axial averaging technique.

### 8.3 Selective analysis

A number of studies limited their analysis to a subset of known lesion prone sites or conditions of shear stress.

- Kharboutly et al [20,21] divided an AVF volume into seven sub-volumes by defining the sections perpendicular to the vascular centreline axis. A set of antipodal points were created along the interior and exterior wall. A selective point comparison analysis of shear stress derived parameters and calcification was performed. A strong association between high TWSSG zones and the presence of the calcification plaques was recorded.
- Rajabi-Jagahrgh et al [32] compared the circumferential distribution of shear stress to a map of IMT at two selective locations of high and low shear stress to find a relationship between low and oscillating shear with maximum IMT.

Both techniques are quantitatively robust, however, limiting their analysis to subsets of data may distort important aspects of the relationship between shear related stimulators of pathological remodelling.

### 8.4 Summary

The circumferential averaging and axial averaging approaches undertaken by researchers to assess lumen changes were quantitatively similar with the later having finer spatial resolution in the axial direction of the vessel. Such quantitative approaches may be apt to quantify the vasodilator induced changes of shear stress but are ill-defined when identifying sites of pathological remodelling particularly IH and its relationship to shear stress. Point by point comparisons or other techniques which directly compare hemodynamic maps with stained or contrast maps will offer a more rigorous analysis for this interaction.

## Limitations and Assumptions of Numerical Simulations

### 9.1 Flow regime and rheology

Determining whether a reconstructed model is realistically reproducing the *in-vivo* hemodynamic environment is difficult to ascertain as there is no benchmark technique for measuring velocities *in-vivo*. The flow within AVFs is characteristically unstable, particularly within the anastomosis and may fall within a transitional to turbulent flow regime. Hence, whether the use of a laminar solution and low resolution meshes are adequate to resolve the flow field needs to be determined.

In all the longitudinal studies the viscosity of blood was kept constant, *in-vivo* it is known that the viscosity of blood decreases during remodelling to promote a return of shear stress to homeostatic values [7]. Only one of the selected articles characterised blood as a non-Newtonian fluid. Despite the high flow environment, regions of low WSS exist within AVFs therefore the influence of blood rheology should be assessed [52].

### 9.2 Boundary conditions

Patient specific reconstructions of the domain of interest need to be coupled with realistic boundary conditions. All studies investigated prescribed a transient flow rate at the arterial inlet with the vast majority applying parabolic velocity profiles, flat or Wormsley profiles. Most studies prescribed a targeted flow rate at the venous outlet and the distal artery outlet based on *in-vivo* measurements. The exceptions prescribed a desired pressure at the venous outlet and recorded a simulated arterial pressure to be within 10% of the mean pressure measured *in-vivo* [8,19]. McGah et al [53] utilised a Windkessel model to set the desired pressure at the venous outlet and recorded venous flows rates to be within 10% of the *in vivo* measurements. However, this boundary condition resulted in a significantly lower pressure drop compared to recent *in-vivo* and computational measurements [54,55].

### 9.3 Distensibility

It is difficult to discern whether the incorporation of distensible wall boundary conditions will improve model accuracy. A recent study by McGah et al [53] found that distensible walls did not reduce very high wall shear stress in AVFs to “normal” levels and concluded that rigid-walled analyses as acceptable for identifying trends in AVFs to establish risk but not acceptable for understanding physical phenomena such as perivascular vibrations (thrills) and bruits within AVFs. The impact of distensibility on the physical phenomena of remodelling rather than the shear stimulus is of more significance as it can potentially limit the level of dilation.

### 9.4 Image reconstruction and mesh sensitivity

Despite the increasing resolution of imaging techniques such as MRI, IVUS and high resolution ultrasound, geometric errors may still persist in the region of 100 μm or more which will affect the accuracy of the absolute WSS values rather than the WSS distribution. The reconstruction of the geometry is also influenced by the level of surface smoothing Excessive smoothing may result in minor shrinkage which would affect the accuracy of absolute WSS values however this level of error is favourable compared to the error resulting from the presence of these imaging artefacts [56].

The spatial and temporal resolution of meshes is another factor which significantly influences the calculation of shear stress and overall accuracy of the solution. A lack of mesh refinement in areas such as the anastomotic junction will result in either under or overestimates the flow field and the absolute values of WSS. Most of the selected articles utilised surface average WSS to determine the spatial and temporal resolution of meshes. A difference of less than 5% between the nominal mesh and finest mesh was deemed to be acceptable by most authors. However, a more robust approach is likely needed to reduce the level of uncertainty.

### 9.5 Summary

The assumption of a Newtonian fluid and rigid wall analyses may overestimate the magnitude of shear stress. Iterative and local refinement of the mesh chosen is necessary to limit these errors. These analyses may overestimate the magnitude of shear stress but won’t skew the variation in the level of shear stress over time. Therefore, such analysis coupled with histological or patient characteristic data are acceptable for determining patient risk of non-maturation. The accuracy of future simulations will depend on the acquisition and reconstruction of patient specific lumen geometry, inlet profiles, flow regime and blood rheology prior to the incorporation of elastic properties of surrounding tissue.

## Vascular Pathology

The endothelium is not only influenced by hemodynamic factors, molecular influences including hypoxia and availability of various soluble factors also influence remodelling. Therefore, it is important to note that the pathology of the vascular vessels may also have a detrimental impact on fistula patency. Johansson et al [51] have shown that radial arteries of ESRD patients have thicker intima and media layers compared to healthy subjects. Some calcification in the intima-media layer has been found in arteries of ESRD patients with uremic toxins cited as contributing factors [57]. Allon et al [58] found that arterial micro-calcification was associated with non-maturing AVFs and reported that arterial or venous intimal hyperplasia was not found in any patients at the time of AVF creation but developed *de novo* after AVF creation. Lee et al [59] have shown extensive calcification in the intima and media of venous segments that were harvested at the time of vascular access surgery. The presence of calcified plaque within the intima-media layer will influence the compliance of the vessel which may limit outward remodelling. Vessel distensibility and elasticity have previously been shown to be better predictors of AVF maturation, compared to preoperative arterial or venous diameter [60–62].

## Discussion

### 11.1 Maturation

It had been generally accepted that remodelling during the maturation process aims to restore shear stress to homeostatic values. However, there is only a partial restoration of shear stress after the maturation process. Shear stress within a mature AVF is not homogeneously distributed over the vessel. High levels of shear stress beyond the physiological range are found to persist in arterial curvatures feeding the anastomosis and within the swing segment of the vein just after the anastomosis due to the velocity jet phenomenon. This results in a non-uniform distribution of high shear stress between the inner and outer walls of this segment. Further away from the anastomosis shear stress levels are found to return to homeostatic values. Significant remodelling occurs within 4–6 weeks post formation with increases in flow and lumen area, after this time point flow rate reduces until remodelling becomes quiescent which may take 3–8 months post formation. From the review it is evident that adequate dilation is a precursor to successful maturation. The creation of the fistulae results in an increase in flow and shear stress within both the artery and vein. The increase or variation of shear stress from the normal level stimulates responsive remodelling. High shear stress tends to stimulate dilation with some elongation and tortuosity.

The increase in lumen area varies along the length of the venous segment due to the non- uniform distribution of shear stress in the segment. In general the increase in lumen area leads to a reduction in shear stress. If the level of shear stress is still beyond the normal level then further dilation is stimulated. When the level of shear stress is within or varies below the physiological range intimal thickening will be primarily triggered to normalise the shear stress level [16–19].

**Fig 6:**
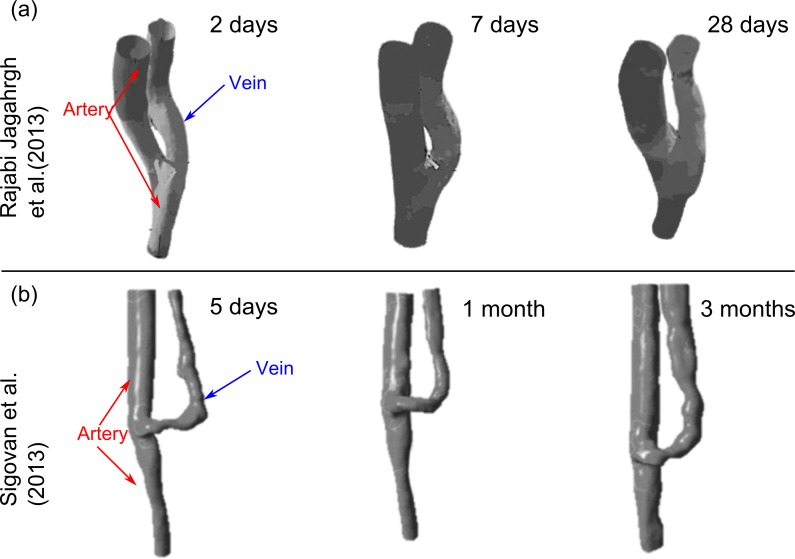
A fistula between the femoral artery and vein in a porcine model and a brachiobasillic fistula in a human patient is shown. For straight configurations there was a larger increase in arterial lumen area compared to venous lumen area at early time points.

In the segment of the vein nearer the anastomosis where it is anchored by suturing the increase in lumen is partially limited [18]. The increase in lumen area reduces the shear stress level but due to the velocity jet phenomenon in this region the level remains high and still varies beyond the physiological range. Dilation and the increase in pressure augment circumferential strain and stimulates medial thickening, the high shear stress present inhibits the magnitude of intimal thickening [16–19]. The balance between vessel thickening and moderate increases in lumen area preserves the lumen calibre of the vessel in this segment. The combination of increased lumen area and intimal and medial thickening over time throughout the vessel is indicative of outward hypertrophic remodelling and successful remodelling. The patterns of shear and remodelling previously described are characteristic of mature fistulae in which there was a large radius of curvature of the venous swing segment. For straight fistulae the patterns are notably different and the outcomes were unfavourable. High shear stresses were found to occupy a large portion of the swing segment. The arterial response may have contributed to impair remodelling of the venous segment. Substantial increases in arterial lumen area in response to high shear stress weakened the impact of venous remodelling as increases in flow resulted in elevated high shear stresses throughout the venous segment [Fig 6].

Supraphysiological shear stress may hinder the beneficial effects of high shear stress as an augmentation in intimal medial thickening is noted. The imbalance between moderate changes in lumen area and substantial IMT reduces the calibre of the vessel and is indicative of inward hypertrophic remodelling which is unfavourable. For straight configurations a greater surface area of the venous segment is subjected to elevated high shear stresses which could denude the endothelium and expose a thrombogenic surface.

### 11.2 Shear stress and Intimal hyperplasia

The remodelling process can alter the lumen geometry at any stage during maturation. Minor changes to geometry can result in acute or chronic changes in shears stress which is an important mediator of vasodilation and vessel remodelling. The occurrence of IH cannot be linked to failure as the severity cannot be quantified. Seemingly intimal thickening attempts to augment shear stress as a part of the remodelling response. If the level of shear is normalised then IH will cease due to re-endothelisation and secretion of atheroprotective genes.

It is evident that the vessel is susceptible to pro-atherogenic factors during the maturation period; what remains to be identified is what switch alters the normal intimal phenotype to one which promotes intimal hyperplasia. A quantitative inverse correlation between shear stress and intimal thickening has been observed within the review with maximal thickening occurring at sites of oscillatory flow. The presence of low shear stress promotes endothelial gene and protein expression of VSMC mitogens which lead to IH. If an unfavourable balance between lumen area and IMT occurs it can lead to a stenosis and would be indicative of impaired remodelling. Despite this quantitative finding and those drawn for other shear related parameters implicated in IH, their interpretations are most likely limited. The variation in experimental conditions amongst studies i.e. time of analysis, classification of IH and variation in data processing makes comparison between theories and findings difficult to establish. Species type and subject age will impact findings as the nominal range of shear and vessel calibres are known to vary. Each of these aspects will need to be addressed and accounted for within future studies.

The rapid aggressive growth of a stenotic lesion *in-vivo* which leads to lumen loss has yet to be accounted by IH alone and has yet to be recreated within these studies. This aggressive remodelling is characterised by a combination of IH and the deposition of platelets and fibrin with an influx and adhesion of inflammatory cells and molecules which result in thrombus remodelling. Therefore, inflammatory markers should be assessed and tracked for future studies.

### 11.3 Prospective work

Future analyses should encompass as many shear based parameters as possible when assessing their influence on endothelial cell function and pathological remodelling. The sensitivity and positive predictive value of each parameter to IMT, IH and thrombus formation should be determined. It is necessary to identify a normal control hence knowledge of the normal functional hemodynamics within maturing AVFs coupled with high resolution imaging or histological data are needed. Quantitative measures of calcification and distensibility will be required to assess the vessel wall and establish normal controls. A number of issues will need to be resolved by future studies prior to this.

#### 11.3.1. Data processing

It is necessary to establish standardised data processing techniques and statistical methods to quantify pathological changes of the vessel wall. The overall level of data reduction through the use of quantitative techniques should be minimal and account for a large subset of data. Visual maps of hemodynamic parameters along with sites or markers of disease should be presented prior to decomposing the data further to find statistically significant relationships. All aspects of hemodynamic parameters should be consider not just mean values as cells are exposed to maximum and minimum values of shear stress derived parameters throughout the cardiac cycle.

#### 11.3.2. Energy conversion

The pressure drop across an AVF may be attributed to energy losses resulting from the presence of turbulent flow which increases resistance. Further *in-vivo* data is needed to confirm such relationships. The presence of turbulent flow may be an important factor in AVF patency, like its nature its role in relation to pathological remodelling may fluctuate. A large pressure drop is needed for enhanced flow and expansive remodelling, however, increased turbulent intensity may disrupt endothelial function and adversely alters its phenotype. The influence of turbulence on endothelial function needs to be delineated further and sites of peak and dissipating turbulent kinetic energy need to be identified to assess energy loss and ensure CFD simulations are depicting the *in-vivo* environment.

## Conclusion

The creation of an AVF initiates a complex cascade of structural remodelling resulting from perturbations in the flow field which generates a non-uniform distribution of shear stress. Variation of shear stress from normal levels will initiate remodelling. The findings of this review suggest that AVF configuration, the reduction of the level of shear stress over time and the balance between dilation and the degree of intimal medial thickening may determine maturation. The later factors are transient aspects of maturation that are not reflected by the single time point measures of flow rate and diameter currently used to assess maturation status.

The various processing techniques used amongst studies reduced the range of shear stress based parameter values over which correlations were sought with the localisation or development of IH. Therefore, the level of data reduction rendered studies incompatible, making it difficult to interpret the pathological response and fully establish the low and oscillatory shear stress theory of intimal hyperplasia development.

If effective diagnostic, therapeutic and surgical strategies are to be developed to promote maturation then standardised models and assessments techniques will need to be adopted by future longitudinal studies to unequivocally clarify the role of shear stress during remodelling. Future analyses should encompass as many shear based parameters as possible, the sensitivity and positive predictive value of each parameter to vasodilation and intimal-medial thickening should be assessed. Robust point or spatial comparison techniques should be employed and categorical factors such as configuration type and vessel pathology should also be accounted for.

## Supporting Information

S1 PRISMA Checklist PRSIMA Checklist. (DOC)

S1 TableWall Shear Stress derived parameters TAWSS, OSI, transWSS, WSSG and TWSSG.(${\vec{\tau }}_{w}$ represents the WSS vector and T represents the period of the cardiac cycle)(DOCX)
