# Supplementary material for: The Role of Shear Stress in Arteriovenous Fistula Maturation and Failure: A Systematic Review
Source: PLoS One. 2015 Dec 30;10(12):e0145795. doi: 10.1371/journal.pone.0145795 (PMC4696682; doi:10.1371/journal.pone.0145795)
Supplement: S1 Table — (τ→w represents the WSS vector and T represents the period of the cardiac cycle) (DOCX) [file pone.0145795.s002.docx]

# Shear stress parameters

A number of wall shear stress derived parameters were used to assess whether they influenced maturation or the prediction of sites of intimal hyperplasia. Three magnitude based shear stress parameters were used and two gradient based parameters were also analysed. Time averaged wall shear stress is calculated by integrating each nodal WSS magnitude over the cardiac cycle. Oscillatory shear index (OSI) is calculated using the formula shown in Eq (**2**). OSI is a dimensionless metric which characterises whether the WSS vector is aligned with the TAWSS vector throughout the cardiac cycle. The transverse wall shear stress (transWSS) calculates the time-average of wall shear stress components perpendicular to the mean flow direction and is defined in Eq (**3**). It quantifies deviations in the direction of the wall shear stress vector throughout the cardiac cycle. The spatial WSS gradient (WSSG) is calculated from the WSS gradient tensor components parallel and perpendicular to the time-averaged WSS vector as outlined in Eq (**4**). The temporal WSS gradient (TWSSG) is simply quantified as the rate of change in WSS magnitude over the cardiac cycle and is outlined in Eq (**5**).

Table S1 Wall Shear Stress derived parameters. TAWSS, OSI, transWSS, WSSG and TWSSG

| **Magnitude based** | Time Averaged WSS | $TAWSS=\frac{1}{T}\int_{0}^{T} \left\vert\vec{\tau}_{w} \right\vert dt$ | (1) |
| --- | --- | --- | --- |
|  | Oscillatory Shear Index | $OSI=0.5\left( 1-\frac{\left\vert\int_{0}^{T} \vec{\tau}_{w} dt \right\vert}{\int_{0}^{T} \left\vert\vec{\tau}_{w} \right\vert dt} \right)$ | (2) |
|  | Transverse Wall Shear Stress | $transWSS=\frac{1}{T}\int_{0}^{T} \left\vert\vec{\tau_{w}}\cdot\left( \vec{n}\times\frac{\int_{0}^{T} \vec{\tau_{w}} dt}{\left\vert\int_{0}^{T} \vec{\tau_{w}} dt \right\vert} \right) \right\vert dt$ | (3) |
| **Gradient based** | WSS Spatial Gradient | $WSSG=\sqrt{\left( \left\vert\frac{\partial\vec{\tau}_{w}}{\partial x} \right\vert\right)^{2}+\left( \left\vert\frac{\partial\vec{\tau}_{w}}{\partial y} \right\vert\right)^{2}+\left( \left\vert\frac{\partial\vec{\tau}_{w}}{\partial z} \right\vert\right)^{2}}$ | (4) |
|  | Temporal WSS Gradient | $TWSSG=\frac{\partial\vec{\tau}_{w}}{\partial t}$ | (5) |

${\vec{\boldsymbol{\tau}}}_{\boldsymbol{w}}$ represents the WSS vector and T represents the period of the cardiac cycle
